# Peritumoral MRI findings and brain herniations in epileptic dogs with prosencephalic brain tumors: a multicentre retrospective study

**DOI:** 10.3389/fvets.2025.1661131

**Published:** 2025-09-19

**Authors:** Marco Tabbì, Domenico Fugazzotto, Chiara Caterino, Simone Minniti, Valeria Toneatti, Giuseppe Barillaro, Simone Minato, Claudia Giannetto, Gerardo Fatone, Francesco Macrì

**Affiliations:** ^1^Department of Veterinary Sciences, University of Messina, Messina, Italy; ^2^Ospedale Veterinario San Francesco, Paese, Italy; ^3^Department of Veterinary Medicine and Animal Production, University of Naples “Federico II”, Naples, Italy; ^4^Clinica Veterinaria San Giorgio (CVSG), Reggio Calabria, Italy; ^5^Clinica Veterinaria San Francesco, Mestre, Italy

**Keywords:** magnetic resonance imaging, brain tumor, brain herniation, epilepsy, canine

## Abstract

Prosencephalic brain tumors (PBTs) are frequently associated with epileptic seizure in dogs, yet the incidence and characteristics of brain herniations (BHs) in this context remain poorly described. This multicentre retrospective study aimed to evaluate the incidence and distribution of BHs and other associated MRI findings in 80 dogs presenting with epileptic seizures secondary to PBTs. MRI studies were assessed for peritumoral edema, lateral ventricular compression, midline shift (MS), subfalcine herniation (SH), caudal transtentorial herniation (CTH), foramen magnum herniation (FMH), and displacement of the quadrigeminal lamina (DQL). Peritumoral edema and lateral ventricular compression were observed in 85 and 77.5% of cases, respectively. MS was observed in 87.5% of cases. SH and CTH were the most common BHs, present in over 75 and 31% of cases, respectively. DQL was observed in 57% of cases, including dogs with tumors anatomically distant from the midbrain. Although tumor volume did not significantly differ between groups, larger lesions were positively correlated with the presence of edema, lateral ventricular compression and laminar displacement. These findings demonstrate that MRI features reflecting mass effect—particularly peritumoral edema, MS, and specific types of BHs—are highly prevalent in dogs with seizure-associated PBTs and may contribute to epileptic seizures generation. The MRI features identified in this study may have prognostic value for potential epileptic seizures development, and should therefore be considered during the clinical evaluation of affected dogs.

## 1 Introduction

Among mammalian species, humans and dogs are commonly reported to develop brain tumors (BTs), with post-mortem observation rates in dogs ranging from 2 to 4.5%. Although the distribution of specific primary BTs varies considerably between studies, approximately 90% of clinically observed primary BTs in dogs are classified as meningiomas (~50%), gliomas (~35%) and choroid plexus tumors (CPT; ~7%) (1–3). The presence of a BT causes various primary and secondary effects leading to intracranial hypertension (ICH) and, ultimately, herniation by a variety of mechanisms (4–6). Brain herniation (BH) refers to shifting of brain structures from their normal compartments within the calvarium (6, 7). The five main types of recognized and commonly described BHs are subfalcine herniation (SH), rostral transtentorial herniation (RTH), caudal transtentorial herniation (CTH), herniation at the foramen magnum (FMH) and herniation through a craniotomy defect (6–8). Among the various types of BHs, SH and CTH have been identified as potential risk factors for tumor-associated epileptic seizures (9). According to Berendt et al., epileptic seizures are “manifestation(s) of excessive synchronous, usually self-limiting epileptic activity of neurons in the brain” that “results in a transient occurrence of signs which may be characterized by short episodes with convulsions or focal motor, autonomic or behavioral features and due to abnormal excessive and/or synchronous epileptic neuronal activity in the brain” (10). Epileptic seizures can be of genetic, idiopathic or structural origin. Structural epilepsy is associated with a wide range of conditions, including inflammatory, infectious, vascular and traumatic diseases, as well as developmental abnormalities, degenerative disorders and neoplastic diseases (11). Epileptic seizures are the most common clinical manifestation of BTs, occurring in approximately 50% of dogs with BTs (3, 9, 12–15). The pathogenesis of tumor-associated seizures remains poorly understood (9, 16–18). Nevertheless, BTs should always be considered as a structural cause of epilepsy in dogs presenting with a first epileptic seizure after 5 years of age, particularly in predisposed breeds (3, 9). Although the association between BTs and epileptic seizure has been frequently reported in dogs, only one study to date has identified specific risk factors for epileptic seizure development in this population (9). The incidence of BHs associated with prosencephalic BTs (PBTs) in dogs is also poorly documented (19). The aim of this multicentric retrospective study was to describe the incidence and type of BHs in dogs with structural epilepsy due to PBTs.

## 2 Materials and methods

The medical records of epileptic dogs referred to the San Francesco Veterinary Hospital and the San Giorgio Veterinary Clinic between January 2022 and December 2024 were retrospectively reviewed. Informed consent was obtained from all owners prior to diagnostic procedures. Dogs were included if they presented with epileptic seizures. Signalment (age, sex, and breed) was collected for each patient. All dogs underwent an MRI of the brain using either a 0.4 Tesla scanner (Hitachi Aperto Lucent, Fujifilm Italia SpA) or a 0.3 Tesla scanner (Hitachi Airis Vento III, Fujifilm Italia SpA). The MRI protocol was performed in accordance with the guidelines proposed by the International Veterinary Epilepsy Task Force (IVETF) for canine brain MRI (20). Each patient was positioned in dorsal recumbency with the head and neck extended. MRI was performed in transverse, sagittal, and dorsal planes using T2-weighted (T2W), fluid-attenuated inversion recovery (FLAIR), T2*-weighted gradient echo (T2* GRE), and T1-weighted (T1W) sequences before and after contrast administration. Gadodiamide (Omniscan^®^, GE Healthcare) was administered intravenously at a dose of 0.015 mmol/kg for post-contrast T1W sequences. All sequences were acquired with a minimum matrix size of 512. T2-weighted 2D images were acquired with slice thicknesses ranging from 2 to 5 mm, repetition times (TR) between 2,952 and 5,500 ms, and echo times (TE) between 90 and 120 ms. FLAIR images were acquired with slice thicknesses ranging from 3 to 5 mm, a TR of 7,000–13,000 ms, and an inversion time of 1800–2,100 ms. Pre-contrast 2D T1W sequences were acquired with slice thicknesses of 2–5 mm, TR of 400–900 ms and TE of 14–26 ms. Post-contrast T1W 3D images were acquired with a slice thickness of 1.2 mm, TR of 30 ms and TE of 12.1 ms. MRIs were interpreted by either a European College of Veterinary Neurology (ECVN) Diplomate or an ECVN Resident under the direct supervision of an ECVN Diplomate. Tumors were classified as intra-axial or extra-axial as previously described (21). Intraventricular tumors were classified as intra-axial, as previously described (22). Tumor location and the following MRI findings were assessed: the presence of midline shift (MS), SH, CTH, FMH, displacement of the quadrigeminal lamina (DQL), lateral ventricular compression (LVC) and peritumoral edema (PE). MS was defined as the displacement of forebrain midline structures on transverse T2W fast spin-echo images. This was assessed by digitally drawing a vertical line from the dorsal sagittal sinus extending along the midline and ending at the midline of the mammillary bodies, as previously described by Oliphant et al. (23). SH was defined as a shift of the cingulate gyrus below the falx cerebri toward the contralateral hemisphere. CTH was defined as unilateral or bilateral caudal displacement of the occipital cortex relative to the osseous cerebellar tentorium. FMH was defined as displacement of the caudal cerebellar vermis into or through the foramen magnum. DQL was defined as a caudal displacement of the caudal colliculus relative to a line between the cerebellar tentorium and the rostral border of the pons. LVC was assessed on T2 FLAIR transverse sequences by comparing the width and symmetry of the lateral ventricles and noting any focal narrowing or collapse. SH, CTH, FMH, DQL and LVC were assessed as previously reported by Bitterman et al. (24). PE was assessed on T2 FLAIR sequences as previously described by Poirier et al. (25).

Volumetric measurements of the tumor (excluding PE) were performed using the planimetry method, as previously described by Thomson et al. (26). The gadolinium-enhanced portion of the tumor mass was manually traced and segmented on each individual slice using designated software, which automatically calculated the area from the traced perimeter. The area measurements were summed and multiplied by the slice thickness and intersection gap to determine the tumor volume (cm^3^) in each of the three anatomical planes. A quantitative analysis of the parametric data was performed on the total number of dogs included regarding tumor location (intra-axial/extra-axial) and the frequency distribution for sex, breed, MS and BHs (MS, SH, CTH and FMH).

A one-way analysis of variance (ANOVA) was then applied to tumor volume, grouping tumors according to the presence of PE only (Group I—G1), LVC only (Group II—G2), PE and LVC (Group III—G3), and neither PE nor LVC (Group IV—G4). Pearson’s correlation and linear regression were used to verify the correlation between tumor volume and animal age. Finally, a point-biserial correlation was used to find the correlation between lamina displacement and tumor volume. The data were analyzed with Statistica 7 (StatSofts, Inc., United States). A *p*-value <0.05 was considered statistically significant.

## 3 Results

**Figure 1:**
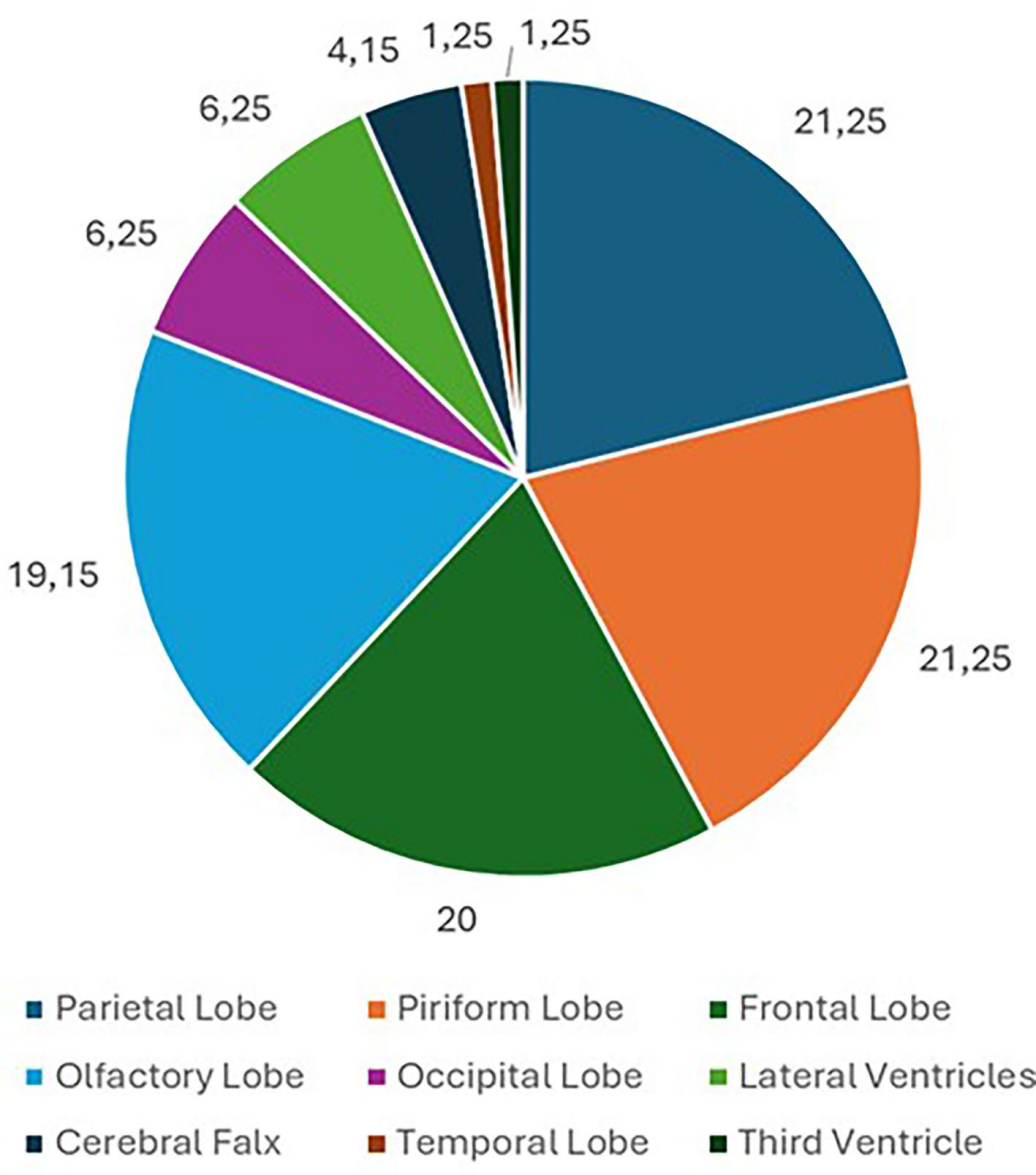
Pie chart of the locations of the prosencephalic brain tumor included in the study.

**Figure 2:**
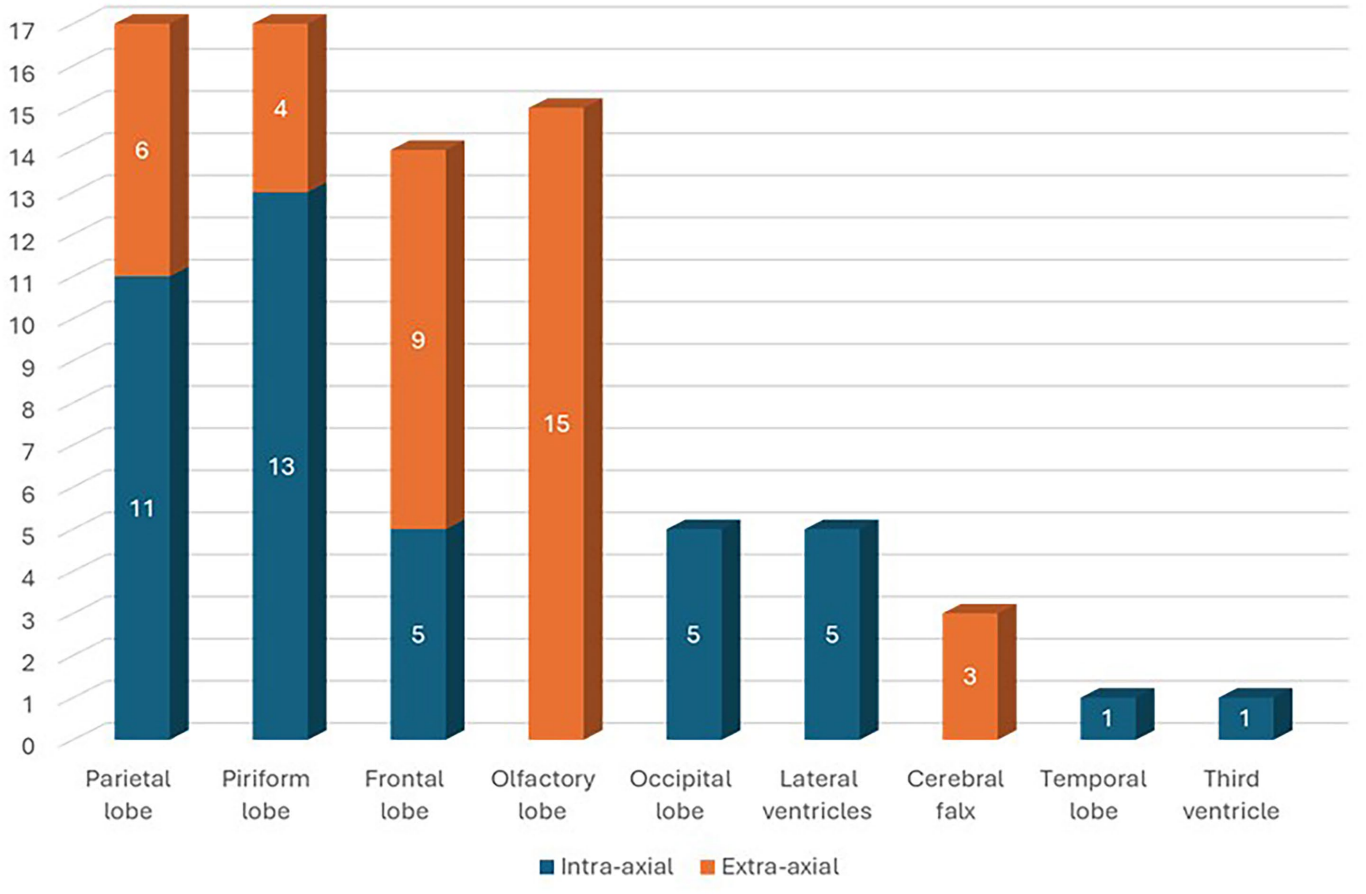
Number of intra-axial and extra-axial tumors divided by locations.

Eighty dogs (*n* = 80) with intracranial tumors met the inclusion criteria. Tumors were located in the parietal lobe (*n* = 17; 21.25%), piriform lobe (*n* = 17; 21.25%), frontal lobe (*n* = 16; 20%), olfactory lobe (*n* = 15; 18.75%), occipital lobe (*n* = 5; 6.25%), lateral ventricles (*n* = 5; 6.25%), cerebral falx (*n* = 3; 3.75%), temporal lobe (*n* = 1; 1.25%), and third ventricle (*n* = 1; 1.25%) (Figure 1). In 43 (53.75%) dogs, the cerebral lesion was intra-axial, while in 37 (46.25%) dogs the lesion was extra-axial. Among parietal lobe lesions, 11 (64.71%) were intra-axial and 6 (35.29%) were extra-axial. Of the piriform lobe lesions, 13 (76.47%) were intra-axial and 4 (23.53%) extra-axial. Of the frontal lobe lesions, 7 (43.75%) were intra-axial and 9 (56.25%) extra-axial. All lesions in the olfactory lobe and cerebral falx were extra-axial. All lesions in the occipital lobe, lateral ventricles, temporal lobe, and third ventricle were intra-axial (Figure 2). For extra-axial lesions, 18 (48.65%) were male and 19 (51.35%) female. For intra-axial lesions, 25 (58.1%) were male and 18 (41.9%) were female. Breed distribution among dogs with intra-axial lesions was: Bichon Frisé (13; 30.23%), mixed breeds (8; 18.6%), Boxer (6; 13.95%), Labrador (3; 6.98%), American Staffordshire Terrier (3; 6.98%), Cane Corso (2; 4.65%), and other breeds (8; 18.61%, each 2.33%). For extra-axial lesions, breeds included: mixed breed (14; 37.84%), German Shepherd (3; 8.11%), Chihuahua (3; 8.11%), Poodle (2; 5.41%), Labrador (2; 5.41%), and other breeds (13; 35.12%, each 2.7%).

**Figure 3:**
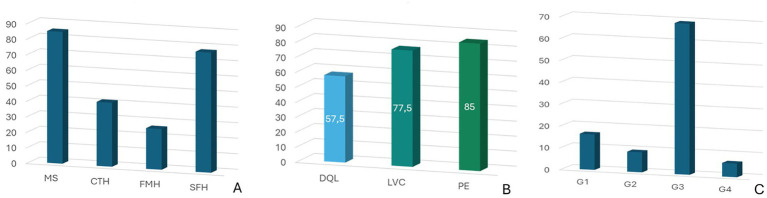
(**A**) Incidence of midline shift (MS) and brain herniations (BHs): subfalcine herniation (SH), caudal transtentorial herniation (CTH), foramen magnum herniation (FMH); (**B**) Incidence of displacement of the quadrigeminal lamina (DQL), lateral ventricular compression (LVC) and peritumoral edema (PE); (**C**) Distribution of tumors according to the presence of PE only (Group I—G1), LVC only (Group II—G2), PE and LVC (Group III—G3), and neither PE nor LVC (Group IV—G4).

**Figure 4:**
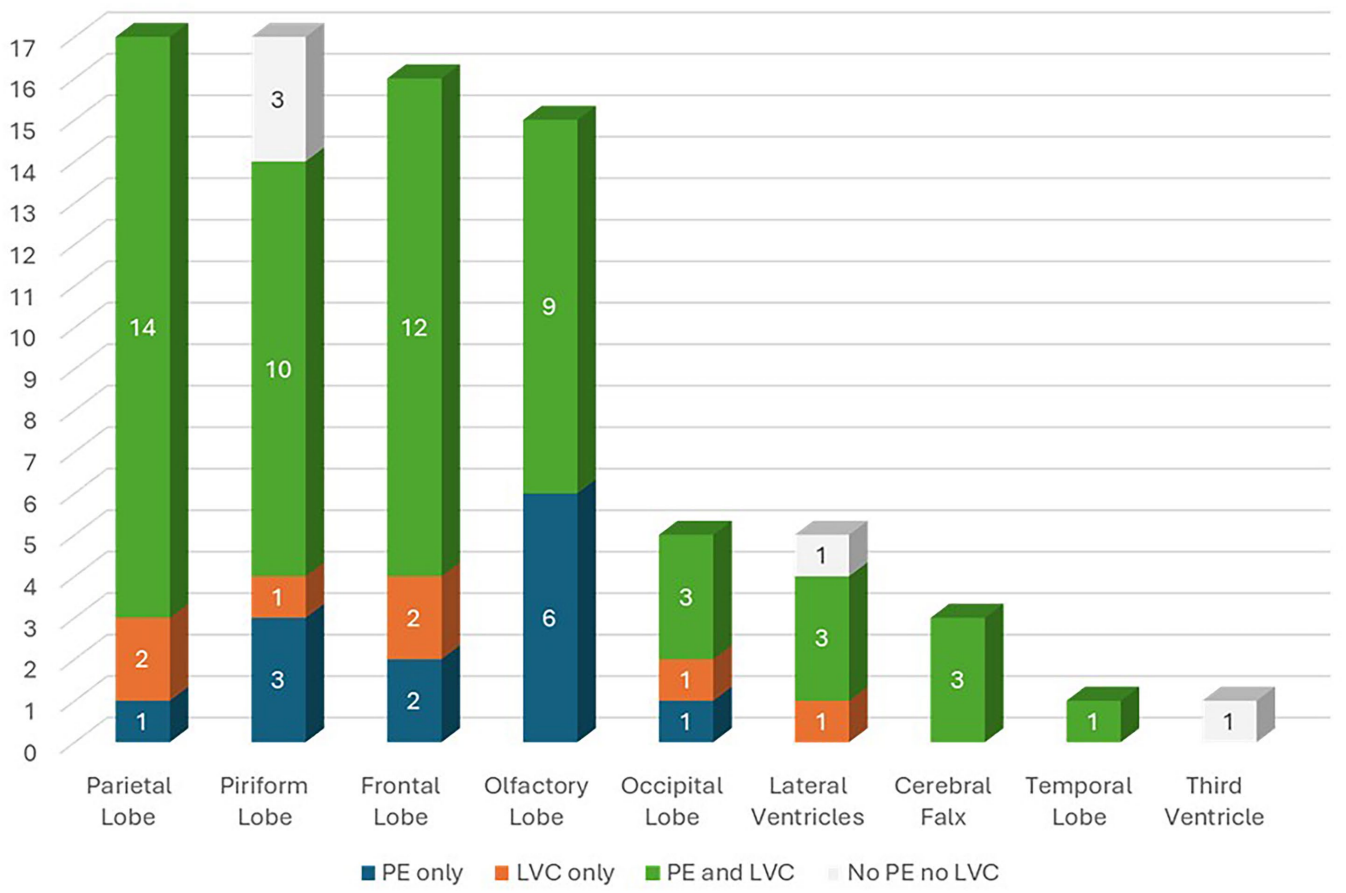
Tumor location according to the presence of peritumoral edema (PE) only (Group I—G1), lateral ventricular compression (LVC) only (Group II—G2), PE and LVC (Group III—G3), and neither PE nor LVC (Group IV—G4).

Overall, 70 dogs (87.50%) had MS, 62 (77.50%) had SH, 33 (41.25%) had CTH and 21 (26.25%) had FMH (Figure 3A). Additionally, 46 dogs (57.50%) showed DQL, 62 (77.5%) showed LVC and 68 (85.0%) showed PE (Figure 3B). Of the dogs included in the study, 13 (16.25%) had PE only (G1), 7 (8.75%) had LVC only (G2), 55 (68.75%) had both PE and LVC (G3) and 5 (6.25%) had neither PE nor LVC (G4) (Figure 3C). Tumor location according to the presence of PE only (G1), LVC only (G2), PE and LVC (G3), and neither PE nor LVC (G4) are summarized in Figure 4.

Lesions causing only PE (G1) had a mean volume of 4.08 ± 3.18 cm^3^ (Table 1). Lesions causing only LVC (G2) had a mean volume of 3.93 ± 1.86 cm^3^ (Table 2). Lesions causing both PE and LVC (G3) had a mean volume of 4.14 ± 2.82 cm^3^ (Table 3). Lesions causing neither PE nor LVC (G4) had a mean volume of 2.32 ± 0.87 cm^3^ (Table 4). One-way ANOVA showed no statistical differences in tumor volume across the groups (G1-G4). Pearson correlation and linear regression analysis revealed no significant correlation between tumor volume and animal age (*p* > 0.05; *r* = −0.18). A positive correlation was found between tumor volume and caudal displacement of the quadrigeminal lamina (*p* = 0.02; *r* = 0.28), particularly in groups G1 (*p* = 0.03; *r* = 0.58) and G2 (*p* = 0.02; *r* = 0.80).

**Table 1:** Location, mean and median tumor volume in cases with magnetic resonance imaging (MRI) signs of peritumoral edema (PE) only (G1).

Extra-axial vs. intra-axial	Tumor quantity/numbers	Prosencephalic location (and total numbers)	Left	Right	Tumor volume
Extra-axial	10/13	Frontal lobe (*n* = 2)	1	1	2.15 ± 1.25
		Piriform lobe (*n* = 1)	0	1	2.49
		Olfactory lobe (*n* = 5)	3	2	5.02 ± 3.97
		Parietal lobe (*n* = 1)	–	–	4.29
		Olfactory-frontal lobe (*n* = 1)	–	–	2.71
Intra-axial	3/13	Piriform lobe (*n* = 2)	0	2	5 ± 4.66
		Occipital lobe (*n* = 1)	1	0	4.26

**Table 2:** Location, mean and median tumor volume in cases with magnetic resonance imaging (MRI) signs of lateral ventricular compression (LVC) only (G2).

Extra-axial vs. intra-axial	Tumor quantity/numbers	Prosencephalic location (and total numbers)	Left	Right	Tumor volume
Extra-axial	1/7	Parietal lobe (*n* = 2)	0	1	2.04
Intra-axial	6/7	Frontal lobe (*n* = 2)	1	1	2.65 ± 0.93
		Piriform lobe (*n* = 1)	0	1	4.37
		Parietal lobe (*n* = 1)	–	–	7.21
		Lateral ventricle (*n* = 1)	–	–	5.00
		Occipital lobe (*n* = 1)	0	1	3.59

**Table 3:** Location, mean and median tumor volume in cases with magnetic resonance imaging (MRI) signs of both peritumoral edema (PE) and lateral ventricular compression (LVC) (G3).

Extra-axial vs. intra-axial	Tumor quantity/numbers	Prosencephalic location (and total numbers)	Left	Right	Tumor volume
Extra-axial	26/55	Frontal lobe (*n* = 7)	4	3	2.04
		Piriform lobe (*n* = 3)	0	3	3.60 ± 2.27
		Olfactory lobe (*n* = 9)	8	1	3.80 ± 1.57
		Parietal lobe (*n* = 4)	2	2	2.34 ± 2.31
		Cerebral falx (*n* = 3)	–	–	3.08 ± 0.43
Intra-axial	29/55	Frontal lobe (*n* = 4)	1	3	4.22 ± 2.93
		Piriform lobe (*n* = 7)	1	6	4.44 ± 2.85
		Parietal lobe (*n* = 9)	8	1	5.09 ± 3.09
		Lateral ventricle (*n* = 3)	–	–	0.55 ± 0.45
		Occipital lobe (*n* = 3)	1	2	3.59
		Temporal lobe (*n* = 1)	–	–	1.89
		Parietal-occipital lobes (*n* = 1)	1	0	12.50

**Table 4:** Location, mean and median tumor volume in cases with no magnetic resonance imaging (MRI) signs of peritumoral edema (PE) or lateral ventricular compression (LVC) (G4).

Extra-axial vs. intra-axial	Tumor quantity/numbers	Prosencephalic location (and total numbers)	Left	Right	Tumor volume
Intra-axial	5/5	Piriform lobe	0	3	1.91 ± 1.72
		III ventricle (*n* = 3)	–	–	2.19
		Lateral ventricle (*n* = 9)	–	–	1.47

## 4 Discussion

Since the adoption of MRI in veterinary practice, the imaging characteristics of BT in dogs have been extensively documented. Histopathological examination remains the only method that provides a definitive antemortem diagnosis of intracranial tumors. However, brain biopsy is technically challenging, carries inherent risks, and is therefore rarely performed as a routine diagnostic procedure (27–29). Consequently, MRI is often employed as a primary technique for the presumptive or preliminary diagnosis of intracranial disease (30, 31). The data obtained from MRI (mass number, origin within the neuroaxis and intrinsic signal appearance) can provide characteristic patterns and support a presumptive diagnosis of the most common BT or at least refine the differential diagnoses. MRI demonstrates a specificity of over 90% for detecting canine BT and a sensitivity of 70–90% for identifying specific tumor types (3, 21, 31, 32). Advances in MRI and quantitative imaging have led to several reviews outlining both common and emerging MRI features of canine BT (30, 33–36). However, the incidence of BHs associated with PBT in dogs is poorly described, especially in epileptic patients (19). Therefore, in this multicentric retrospective study, we described the incidence and type of BHs in epileptic dogs with PBT.

Intracranial tumors can be classified by anatomical location (supratentorial, subtentorial, basilar, etc.) or by their origin within the neuroaxis (extra-axial, intra-axial or intraventricular). Extra-axial tumors originate from structures outside the neural axis (e.g., meninges), whereas intra-axial tumors arise from within the neural parenchyma (30). Although often included in a separate category or described as extra-axial, in our study we classified six intraventricular tumors (6/80) as intra-axial, as previously described (22). Our study found a slightly higher prevalence of intra-axial tumors (43/80, 53.75%) compared to extra-axial tumors (37/80, 46.25%), which is consistent with the findings reported by Snyder et al. (1). However, other studies have reported a predominance of extra-axial tumors (2, 3, 9). This discrepancy may reflect differences in breed representation, the exclusive inclusion of epileptic dogs, or the classification of intraventricular tumors within either category. In human medicine, the occurrence of epileptic seizures associated with BT is strongly correlated with tumor type and has been suggested to be more frequent in intra-axial tumors such as oligodendrogliomas and astrocytomas, according to one study (37). Although a statistically significant relationship between tumor type and epileptic seizure development has not yet been demonstrated in veterinary medicine, oligodendrogliomas have been shown to more frequently predispose dogs to epileptic seizures compared to other BT (1). The inclusion in our study of dogs presenting epileptic seizures secondary to PBT may therefore explain the slight predominance of intra-axial neoplasms over extra-axial ones. A statistically significant correlation between breed, body weight, age and incidence of brain tumors has been reported, with a higher prevalence of extra-axial tumors (mostly meningiomas) in Golden Retrievers, mixed-breed dogs, Miniature Schnauzers and Rat Terriers, and intra-axial tumors (astrocytomas, oligodendrogliomas and unspecified gliomas) in Boston Terriers, Bullmastiffs, English and French Bulldogs and other brachycephalic breeds (1, 2, 38). In our study, Bichon Frises were the breed most affected by intra-axial tumors (13/43, 30.23%), while mixed-breed dogs had the highest incidence of extra-axial tumors (14/37, 37.84%). Our results are consistent with previous reports suggesting a breed predisposition to specific tumor types (2, 3). Regarding sex distribution, 25 of the 43 dogs (58.1%) with intra-axial tumors (43/80) were male, while 18 (41.9%) were female. In the group with extra-axial tumors (37/80), 18 dogs (48.65%) were male and 19 (51.35%) were female. These findings suggest a mild male predominance in intra-axial tumors, while extra-axial lesions appeared to be slightly more frequent in females, although no statistically significant association was assessed in this study.

The causal relationship between intracranial neoplasms and epileptic seizure has been widely reported in dogs (1, 9, 18). Lesion localization has been identified as an important risk factor for the development of epileptic seizures in dogs, particularly in regions with lower epileptogenic threshold due to their connections with cortical and subcortical structures involved in the initiation and propagation of epileptic seizures (9). Therefore, analyzing the distribution of different PBT in epileptic dogs could provide supplementary information, aiding the identification of potential risk factors. In our study, the tumor distribution was higher in the parietal (21.25%), piriform (21.25%), and frontal (20%) lobes. In dogs with PBT, both primary localization and secondary invasion of the frontal, piriform and temporal lobes have been associated with a higher epileptic seizure risk, even in the absence of PE or marked mass effect (9, 31). The high incidence of PBT in the parietal lobe observed in our study may suggest a primary epileptogenic role for this area in dogs, similar to what has been reported for other forebrain regions (9).

Acute clinical deterioration observed in animals with BT and ICH is often the result of a combination of various mechanisms including direct mass effect, PE, obstructive hydrocephalus, cerebral ischemia or hemorrhage, and finally BH (5, 6). MS is a recognized indicator of mass effect and increased intracranial pressure, and has been correlated with an increased mortality rate and a worsening of the neurological condition in dogs with BT (24, 39). A previous study observed an MS rate of at least 20% in 40% of dogs with extra-axial tumors (particularly meningiomas) and 36% of dogs with intra-axial tumors (particularly gliomas) (40). More recently, another study reported an MS prevalence of 51.95% in a cohort of 77 dogs (39). In our study MS was observed in 87.5%% of cases, confirming the high prevalence of this sign. Brain herniation is a major complication of PBT and an important risk factor for the development of epileptic seizures in dogs (9, 19). SH was frequently observed (77.5%), suggesting a potential role in the pathogenesis of epileptic seizures. Along with CTH, SH is a significant risk factor for tumor-associated structural epilepsy that can be identified on MRI scans. Higher rates of epileptic seizures in cases of SH and CTH may be explained by ischemia due to increased intracranial pressure and reduced cerebral perfusion (6, 9). SH can be epileptogenic also due to its effect on the cingulate gyrus, which lies immediately adjacent to the falx cerebri and is therefore particularly vulnerable to compression or displacement in this type of BHs (41). Evidence from veterinary neuroimaging further supports the cingulate region’s role in canine epilepsy (42). Structural MRI studies have also shown a significant reduction in cingulate gyrus volume in affected dogs, reinforcing its role in seizure generation. These findings help explain why compression of the cingulate during SH could lower the seizure threshold (43). Experimental and clinical studies in dogs have further shown that structural or functional alterations of the cingulate cortex, including cortical atrophy, gliosis, and astrocytosis, are associated with spontaneous seizures and drug-resistant epilepsy (44, 45). In our study, CTH and FMH were observed in 41.25 and 26.25% of cases, respectively. These percentages are higher than those reported in a previous study, in which CTH and FMH were observed in 20 and 4.4% of cases, respectively (19). Conversely, SH was the most common BH in that study (62%), a finding consistent with our data. In another study among 88 dogs, 40.91% had CTH alone, 19.32% had FMH alone, and 39.77% had both with Mixed-breed dogs, Boxers, Boston Terriers and Golden Retrievers being the most frequently affected breeds (7). Mixed breeds were also the most affected by CTH in our population (54.5%), while Bichon Frises had a higher incidence of FMH (28.6%). These data aligned with previous studies that have associated these BHs with a poor prognosis, especially in cases where brainstem compression occurs (7, 19).

Although the pathogenesis of tumor-associated epileptic seizures is poorly understood (16, 17, 46), the majority of BT originate from non-neuronal cells that lack intrinsic epileptogenic properties. Therefore, the development of epileptic seizures may depend on the effects on the surrounding neuronal tissue. Several pathophysiological mechanisms have been proposed for tumor-associated epileptogenesis. These include local cerebral ischemia, isolation and denervation of cortical areas, neuronal, axonal and synaptic plasticity, and others (9, 46). Increased brain excitability is primarily caused by PE and related ionic, pH and extracellular osmolarity alterations (5, 9, 17). Despite that the pathophysiology of PE is still not fully understood, vasogenic edema is widely accepted as the predominant form, resulting from blood–brain barrier disruption and increased vascular permeability (3, 25, 30, 47–50). In both human and veterinary medicine, vascular endothelial growth factor (VEGF) has been implicated in the development of vasogenic edema, particularly in meningiomas (25, 50–54). In our study, PE was observed in 85% of dogs, supporting findings from Cherubini et al. who reported an 82% incidence in extra-axial BT (49) and from Sturges et al., who observed 94% incidence (50). Therefore, the high prevalence of PE in our population likely reflects the underlying vasogenic mechanisms commonly associated with both intra- and extra-axial PBT. The location of peri-lesional T2 hyperintensity, used to assess PE, was found to be more predictive of epileptic seizure onset than the actual degree of edema (9). This is consistent with the idea that different brain regions have different thresholds of excitability (46).

In humans, displacement of deep structures such as the quadrigeminal lamina is associated with altered consciousness, visual symptoms and poor prognosis (55). In dogs, compression of these areas may explain behavioral, visual and motor changes, particularly in cases of expansive parieto-occipital tumors. DQL can result from several conditions, including increased intracranial pressure (ICP), vascular abnormalities, quadrigeminal cistern arachnoid cysts and PBT (24, 56–58). Arachnoid cysts of the quadrigeminal cistern can compress the lamina, and an association with epileptic seizures in dogs has been reported (57–60). In our study, DQL was identified in 57.5% of cases and could therefore be considered both a prognostic sign of disease and an additional risk factor for the development of epilepsy associated with PBTs, similar to other BHs. We also found a positive correlation between tumor volume and DQL (*p* = 0.02; *r* = 0.28), particularly in lesions causing PE only (G1) (*p* = 0.03; *r* = 0.58) and LVC only (G2) (*p* = 0.02; *r* = 0.80), both of which may contribute to DQL. Although tumor location is known to influence epileptic seizure onset (37), many of the PBT in our study were not in direct proximity to the quadrigeminal lamina. Therefore, our data suggest that larger lesions associated with PE and LVC may cause DQL even when distant from the quadrigeminal lamina, potentially contributing to epileptic seizure generation.

This study has several limitations related to its retrospective nature. Although MRI features were carefully evaluated and classified according to current standards, the absence of histopathological confirmation prevented definitive tumor classification and grading, which would have provided valuable information for correlating imaging findings with tumor type and biological behavior. Secondly, the MRI scans were acquired using low-field equipment (0.25 T), which, although commonly used in veterinary practice, may have lower sensitivity than high-field MR in detecting less extensive parenchymal or structural alterations. Furthermore, the 2–5 mm slice thickness may have been too large. Thinner slices would have allowed for more precise evaluations. Finally, an increased sample size would provide further validation of the results.

## 5 Conclusion

This multicenter retrospective study describes the prevalence and distribution of MR features associated with PBT in epileptic dogs. Tumor affecting the parietal lobe were particularly frequent, suggesting a primary epileptogenic role for this area due to its connections with cortical and subcortical networks, similar to other forebrain structures. Peritumoral alterations—including PE, LVC, DQL, MS, and SH—were commonly observed and appear to contribute to lowering the seizure threshold. Despite the absence of significant differences in tumor volumes between the groups, a positive correlation was found between tumor volume and the presence of PE, LVC, and DQL, indicating that larger lesions may cause DQL even when distant from the quadrigeminal lamina, potentially contributing to seizure generation. These findings emphasize the interplay between tumor location, volume, and secondary structural effects, and suggest that specific peritumoral MRI features mayserve as a valuable clinical and prognostic markers for dogs with seizures associated to PBT. Despite these insights, the study has limitations, including its retrospective design, the lack of histopathological confirmation, the use of low-field MRI (0.25 T) with 2–5 mm slice thickness, and a relatively limited sample size. Nonetheless, these results provide important information for risk stratification and clinical management. Future prospective studies with larger cohorts and higher-resolution imaging are needed to validate these associations and further clarify how peritumoral changes contribute to seizure generation.

## Data Availability

The original contributions presented in the study are included in the article/supplementary material, further inquiries can be directed to the corresponding author.
